# TIMM17A overexpression in lung adenocarcinoma and its association with prognosis

**DOI:** 10.1038/s41598-024-59526-1

**Published:** 2024-04-17

**Authors:** Lili Miao, Dejun Wu, Hongyu Zhao, Aiwei Xie

**Affiliations:** 1 Department of Respiration, YiZheng People’s Hospital, YiZheng, Jiangsu China; 2 Department of Nephrology, YiZheng People’s Hospital, YiZheng, Jiangsu China

**Keywords:** TIMM17A, Lung adenocarcinoma, Prognosis, Biomarker, Cancer, Medical research, Oncology

## Abstract

Lung adenocarcinoma (LUAD), a leading cause of cancer-related mortality worldwide, demands a deeper understanding of its molecular mechanisms and the identification of reliable biomarkers for better diagnosis and targeted therapy. Leveraging data from the Cancer Genome Atlas (TCGA), the Clinical Proteomic Tumor Analysis Consortium (CPTAC), and the Human Protein Atlas (HPA), we investigated the mRNA and protein expression profiles of TIMM17A and assessed its prognostic significance through Kaplan–Meier survival curves and Cox regression analysis. Through Gene Set Enrichment Analysis, we explored the regulatory mechanisms of TIMM17A in LUAD progression and demonstrated its role in modulating the proliferative capacity of A549 cells, a type of LUAD cell, via in vitro experiments. Our results indicate that TIMM17A is significantly upregulated in LUAD tissues, correlating with clinical staging, lymph node metastasis, overall survival, and progression-free survival, thereby establishing it as a critical independent prognostic factor. The construction of a nomogram model further enhances our ability to predict patient outcomes. Knockdown of TIMM17A inhibited the growth of LUAD cells. The potential of TIMM17A as a biomarker and therapeutic target for LUAD presents a promising pathway for improving patient diagnosis and treatment strategies.

## Introduction

Lung adenocarcinoma (LUAD), an insidious disease originating from malignant cells in the lung tissue, imposes a significant global burden, with approximately 2 million new diagnoses and 1.6 million deaths reported annually^1^. Treatment modalities for LUAD, ranging from surgical resection to advanced therapies like targeted therapy, have shown promise. Surgical resection stands as a cornerstone, providing a survival advantage for early-stage cases, while advanced stages often necessitate radiotherapy and chemotherapy^2–7^. The emergence of targeted therapy has revolutionized treatment by honing in on specific molecular phenotypes, leading to improved outcomes^8–11^. Despite progress, some patients resist current treatments. Exploring genetic markers, especially key LUAD genes, is crucial for personalized therapies and improved outcomes.

Mitochondria are organelles that serve as crucial energy centers within cells, facilitating cellular metabolism, apoptosis, and other vital cellular processes^12^. One key factor in mitochondrial function is the import of proteins into the mitochondrial matrix, which is facilitated by the mitochondrial inner membrane translocase (TIM) complex. A significant component of this complex is Translocase of Inner Mitochondrial Membrane 17A (TIMM17A), which has been shown to play an essential role in mitochondrial function^13^. Interestingly, recent research has highlighted the potential role of TIMM17A in breast cancer, where its expression is upregulated and associated with poor pathological and clinical outcomes^13^. Moreover, TIMM17A has been identified as a potential biomarker for breast cancer and has been found to promote the proliferation and migration of breast cancer cells^14^. Furthermore, the regulation of TIMM17A is closely tied to cellular stress, where stress-regulated translational attenuation adapts to the import of mitochondrial proteins through the degradation of TIMM17A, which is essential for the maintenance of mitochondrial DNA^15^. Additionally, the PI3K/AKT signaling pathway, which is implicated in tumorigenesis and cancer progression, has been found to be involved in the action of TIMM17A and its role in breast cancer^12^. Recent studies have also delved into the genetic and structural characteristics of TIMM17A and the TIM complex, revealing multiple translocases that maintain mitochondrial function and uncovering the complex organization of mammalian mitochondrial presequence translocases^16,17^.

This study adopted a comprehensive approach to elucidate the role of TIMM17A in LUAD. Our research commenced with an examination of TIMM17A expression levels in LUAD, utilizing data from the Cancer Genome Atlas (TCGA), Clinical Proteomic Tumor Analysis Consortium (CPTAC), and Human Protein Atlas (HPA) to analyze both mRNA and protein expression profiles. The analysis was designed to understand the correlation between TIMM17A expression and various pathological features of LUAD, primarily using the TCGA database. This foundational work set the stage for subsequent survival analysis, including Kaplan–Meier curves and Cox regression analysis, aimed at validating the prognostic significance of TIMM17A expression in LUAD. To further our exploration, a nomogram model was developed for prognosis prediction, and Gene Set Enrichment Analysis (GSEA) was employed to uncover potential mechanisms by which TIMM17A might influence LUAD progression. The study's design also incorporated the collection of pathological and prognostic data from 85 LUAD patients to assess the impact of TIMM17A expression on patient outcomes, alongside in vitro experiments with A549 cells to examine the effects of TIMM17A on cell proliferation.This approach underscores our commitment to investigating TIMM17A's utility as a diagnostic and prognostic marker for LUAD. It is pivotal to highlight that our study is designed to lay the groundwork for further detailed analysis necessary to fully understand the biomarker potential of TIMM17A in LUAD and to validate its applicability in clinical settings.

In summary, these research findings propose an intriguing possibility that TIMM17A could serve as a valuable diagnostic and prognostic factor for LUAD. However, it is essential to emphasize that comprehensive elucidation of the potential mechanisms underlying TIMM17A as a biomarker in LUAD and the validation of its clinical utility require further in-depth investigation.

## Methods and materials

### Data collection

This study comprehensively utilizes LUAD transcriptomic datasets in conjunction with clinical data from the TCGA database. (https://portal.gdc.cancer.gov/) for a thorough investigation into LUAD. To enhance this analysis, the researchers sourced a comprehensive cancer transcriptome dataset from the XENA database (https://xenabrowser.net/datapages/). Additionally, to comprehensively assess survival outcomes, we acquired Progression-Free Interval (PFI), Disease-Free Interval (DFI), and Disease-Specific Survival (DSS) data for LUAD from the same XENA database^18^.

### Survival rate and ROC analysis

We used the 'survival' and 'survminer' packages for Kaplan–Meier survival curve analysis and survival ROC analysis within the R software framework (ver. 4.1.3). Through these analytical procedures, we were able to derive an AUC value that served as a reliable metric for evaluating the accuracy of our predictions. The ROC curves were generated using analytical tools available through the xiantao academic website (https://www.xiantao.love/), facilitating precise and effective visualization of our diagnostic predictions^19^.

### HPA database and CPTAC database

In the present inquiry, an approach was employed, whereby immunohistochemical chips derived from the Human Protein Atlas (HPA) database^20^ (located at https://www.proteinatlas.org/) were utilized in conjunction with protein expression data obtained from the Clinical Proteomic Tumor Analysis Consortium (CPTAC), which was sourced from the University of Alabama Cancer Database (UALCAN) (found at https://ualcan.path.uab.edu/index.html^21^.

### Nomogram model

To predict the 1-year, 3-year, and 5-year overall survival (OS) rates for patients with lung adenocarcinoma (LUAD), nomogram models were carefully constructed using the "rms" package in R statistical software (version 4.1.3). These models, designed to consider various factors affecting survival rates, were then evaluated for their reliability and accuracy through the plotting of calibration curves. This step allowed for a detailed assessment of the model's predictions against observed outcomes, ensuring the models' accuracy and reliability^22^.

### Gene set enrichment analysis (GSEA)

To thoroughly investigate the multifaceted and complex functions of TIMM17A within intricate signaling pathways, computational tool GSEA software was employed, utilizing the TCGA LUAD dataset. In order to ensure the robustness and significance of the study findings, 1000 sequencing tests were conducted. Through these numerous tests, statistically significant pathways with a remarkable False Discovery Rate (FDR) q value of less than 0.05 were pinpointed and extensively analyzed^23^.

### Univariate and multivariate Cox regression analysis

To thoroughly investigate the independent factors influencing the overall survival (OS) rate in patients with lung adenocarcinoma, we applied standard univariate and multivariate Cox regression analyses. This approach was implemented to meticulously scrutinize the impacts of pivotal variables, namely age, gender, T, N, M, and TIMM17A expression levels, on the prognosis of LUAD, with utmost scrupulousness^19^.

### Quantitative reverse transcription polymerase chain reaction (qRT-PCR)

Complementary DNA (cDNA) was synthesized using 20 μl of HiScript III RT SuperMix for qPCR (+gDNA wiper) (Vazyme, Nanjing), following the manufacturer's instructions. qRT-PCR was then performed as per the kit's guidelines to ensure accuracy and reproducibility^24^. In order to evaluate the expression levels of the target genes, specific primer sequences were utilized: GAPDH Forward: TGCACCACAACTGCTTAGC; GAPDH Reverse: GGCATGGACTGTGGTCATGAG; TIMM17A Forward: GCTCCACAGTTAGGAGGTAGC; TIMM17A Reverse: CTCCCGTTAAGGCACCACTT. To validate the accuracy and consistency of our findings, all experiments were repeated three times.

### Patient samples

85 pairs of fresh tumor and paracancerous tissue samples from patients with LUAD were obtained from YiZheng People's Hospital. None of the patients had undergone radiotherapy, chemotherapy, or immunotherapy prior to sample collection. This study received approval from the hospital's ethics committee, and written informed consent was provided by all participating patients (No. 2019-27).

### Cell culture and transfection

A549 cells were purchased from Pricella (Wuhan, China). The cells were cultured in Ham's F-12k medium supplemented with 10% fetal bovine serum (FBS) and 1% penicillin/streptomycin. The culture was maintained at 37 °C in a incubator with 5% CO_2_. After passaging cells and culturing them in a six-well plate for 24 h, transfection was performed when the cell density reached 60–70%. Transfection of TIMM17A siRNA (Ruibo, Guangzhou) was carried out using Lipofectamine 3000 transfection reagent (Invitrogen, USA). Cells were collected 48 h post-transfection to extract RNA for assessing transfection efficiency, all experiments performed in triplicate^25^.

### Cloning experiment and edu assay

Cells were collected 48 h post-transfection, resuspended after centrifugation, and counted using a cell counting plate. Cell density was adjusted, and 500 cells were seeded per well in a six-well plate. Each well was supplemented with 2 ml of complete medium. The medium was changed every 3–5 days, and the cells were cultured for approximately 2 weeks. After removing the six-well plate, the culture medium was aspirated, and cells were fixed with 4% paraformaldehyde at room temperature for 30 min. After fixation, crystal violet staining was performed for 5 min, followed by washing with PBS for 2–3 times. The plates were air-dried, observed, and photographed. Edu experiments were conducted following the instructions of the Edu-488 Cell Proliferation Assay Kit (Bi Yun Tian, Shanghai)^26^, with all experiments performed in triplicate.

### Statistical analysis

In the conduct of this empirical inquiry, we employed an assortment of statistical techniques to dissect information in various contexts. For data that resided within a dyadic framework, we harnessed the analytical power of the independent Wilcoxon test, thereby securing a statistically significant reckoning. In the event that data accrued from a tripartite or quadrilateral schema, we opted to implement the Kruskal–Wallis test, which furnished a statistically significant determination. We conducted univariate survival analysis using Kaplan–Meier survival analysis and the log-rank test to assess statistical significance. Additionally, for multivariate survival analysis, we employed the Cox regression model, considering variables with a *p* value below the critical threshold of 0.05 as statistically significant.

### Ethics statement

This study was approved by the Institutional Research Ethics Committees of the YiZheng People’s Hospital. We confirmed that all methods were carried out in accordance with relevant regulations and written informed consent was obtained from patients.

## Result

### TIMM17A is abnormally up-regulated in LUAD

**Figure 1:**
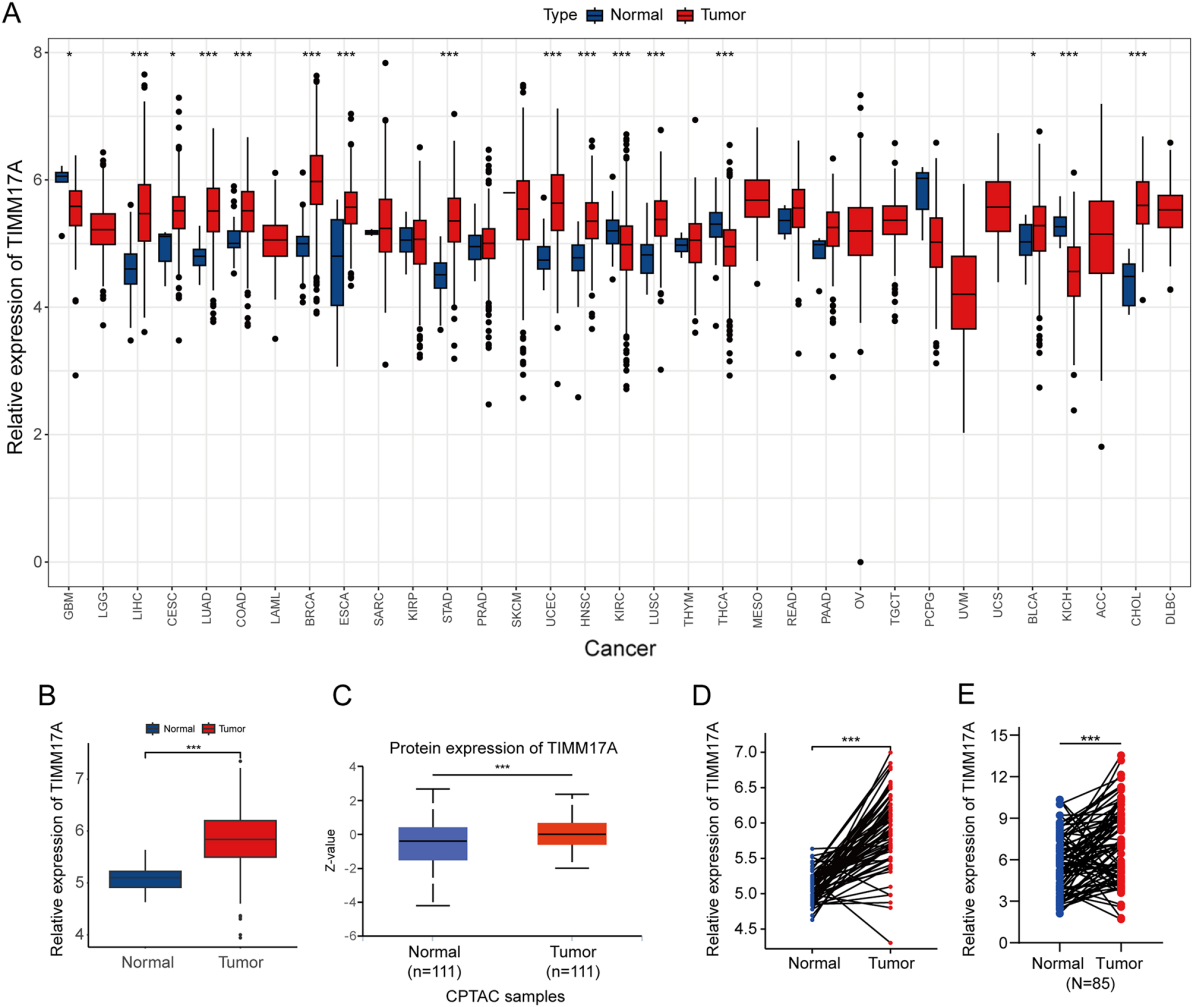
TIMM17A was abnormally up-regulated in LUAD. (**A**–**B**) TIMM17A expression is abnormally down-regulated in GBM, THCA, KICH, and KIRC, TIMM17A expression is abnormally up-regulated in LIHC, CESC, LUAD, COAD, BRCA, ESCA, STAD, UCEC, HNCS, LUSC, BLCA, and CHOL. (**C**) TIMM17A protein expression is abnormally up-regulated in LUAD tissues according to the CPTAC database. (**D**) TIMM17A expression is aberrantly up-regulated in LUAD tissues based on paired analysis of TCGA LUAD dataset. (**E**) TIMM17A expression is similarly up-regulated in LUAD tissues compared to normal lung tissues based on qRT-PCR analysis of paired tissue samples.

**Figure 2:**
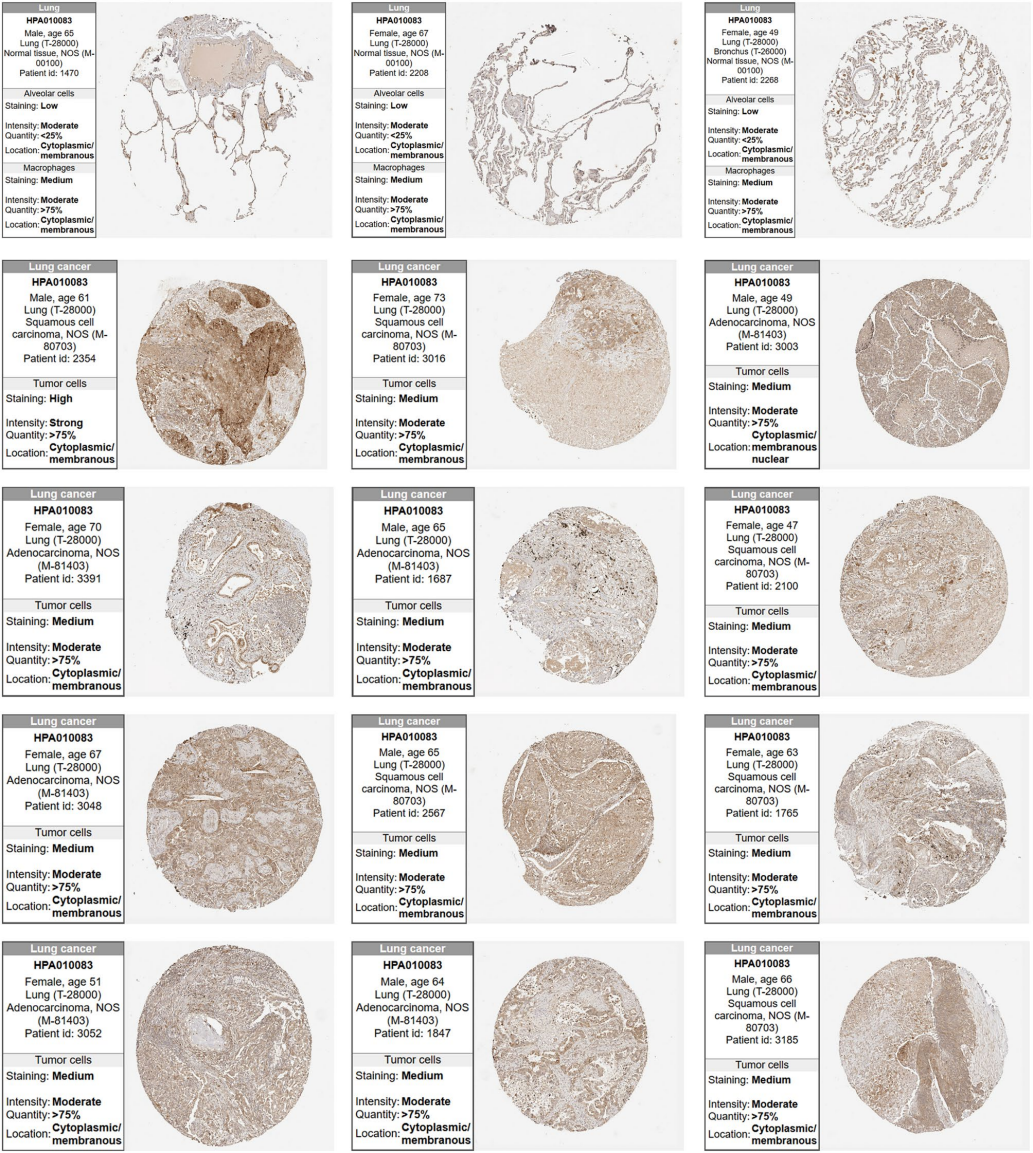
TIMM17A protein expression in LUAD. TIMM17A protein expression is abnormally elevated in LUAD according to the HPA database.

To investigate the expression levels of TIMM17A, we analyzed transcriptome data from 33 types of cancer in the TCGA database.Among these, the tumor data for LGG, LAML, MESO, OV, TGCT, UVM, UCS, ACC, and DLBC lack corresponding normal control groups. In the remaining 24 types of tumors, TIMM17A expression demonstrated varying patterns. It was downregulated in only four tumor types: GBM, THCA, KICH, and KIRC. Aberrant expression of TIMM17A was observed in 12 different cancers, including LIHC, CESC, LUAD, COAD, BRCA, ESCA, STAD, UCEC, HNCS, LUSC, BLCA, and CHOL. However, no expression differences of TIMM17A were observed in SARC, KIRP, PRAD, SKCM, THYM, READ, PAAD, and PCPG. (Fig. 1A,B). Furthermore, in LUAD tissues, abnormal upregulation of TIMM17A protein expression was validated using the CPTAC database (Fig. 1C). Subsequently, paired analysis of TCGA LUAD dataset consistently showed aberrant upregulation of TIMM17A expression in LUAD tissues (Fig. 1D). To validate these findings, we collected 85 pairs of LUAD tissues and their corresponding normal lung tissues. qRT-PCR confirmed the abnormal upregulation of TIMM17A expression in LUAD tissues (Fig. 1E). Additionally, a thorough examination of the HPA database corroborated these observations, demonstrating a similarly elevated expression of TIMM17A protein in LUAD (Fig. 2A). The aforementioned results highlight the significant upregulation of TIMM17A expression in LUAD.

### TIMM17A is associated with poor prognosis in LUAD.

**Figure 3:**
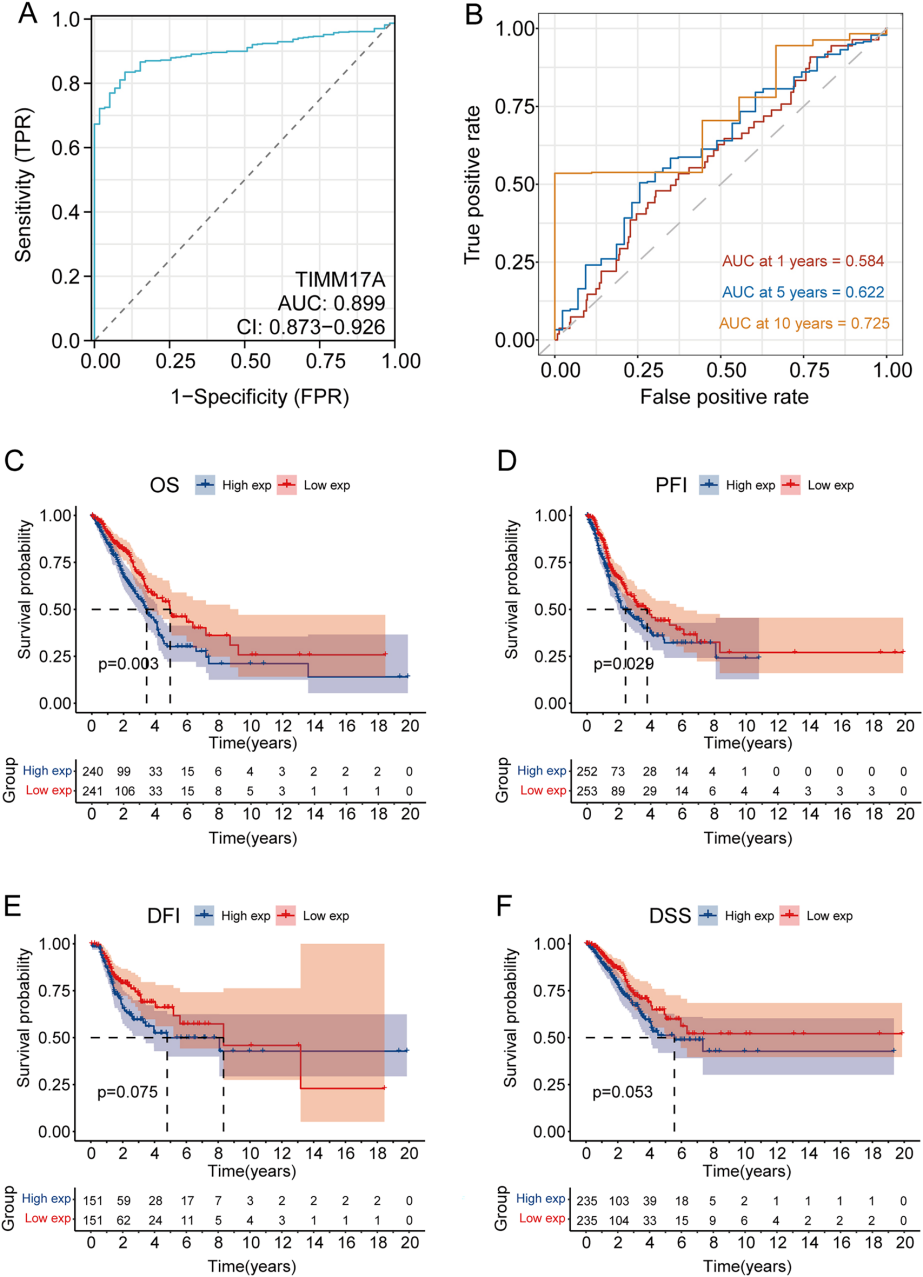
TIMM17A is associated with poor prognosis in LUAD. (**A**) The TIMM17A gene exhibits high proficiency in identifying LUAD with an AUC of 0.899. (**B**) TIMM17A shows substantial potential in diagnosing advanced LUAD with an AUC of 0.725 at year 10 based on time-dependent ROC curve analysis. (**C**) Elevated TIMM17A expression is positively correlated with impaired OS of LUAD patients. (**D**) Elevated TIMM17A expression is positively correlated with impaired progression-free interval (PFI) of LUAD patients. (**E**) No significant statistical difference was observed between TIMM17A expression and DFI of LUAD patients. (**F**) No significant statistical difference was observed between TIMM17A expression and DSS of LUAD patients.

Given the aberrant expression of TIMM17A in LUAD, we conducted a thorough investigation into its relationship with LUAD prognosis. Initially, the TIMM17A gene demonstrated notable effectiveness in LUAD detection, evidenced by an area under the curve (AUC) of 0.899 (Fig. 3A). Subsequent analysis using time-dependent receiver operating characteristic (ROC) curves revealed TIMM17A's significant diagnostic potential for advanced LUAD, with an AUC of 0.725 at 10 years (Fig. 3B). Additionally, there was a positive correlation between increased TIMM17A expression and reduced OS and PFI in LUAD patients (Fig. 3C,D). While there were no significant statistical differences in DFI and DSS associated with TIMM17A expression (Fig. 3E,F), a trend towards poorer prognosis with higher TIMM17A levels was observed. These findings suggest that TIMM17A could be a viable prognostic biomarker for LUAD.

### Relationship between TIMM17A expression and clinical features in patients with LUAD

**Figure 4:**
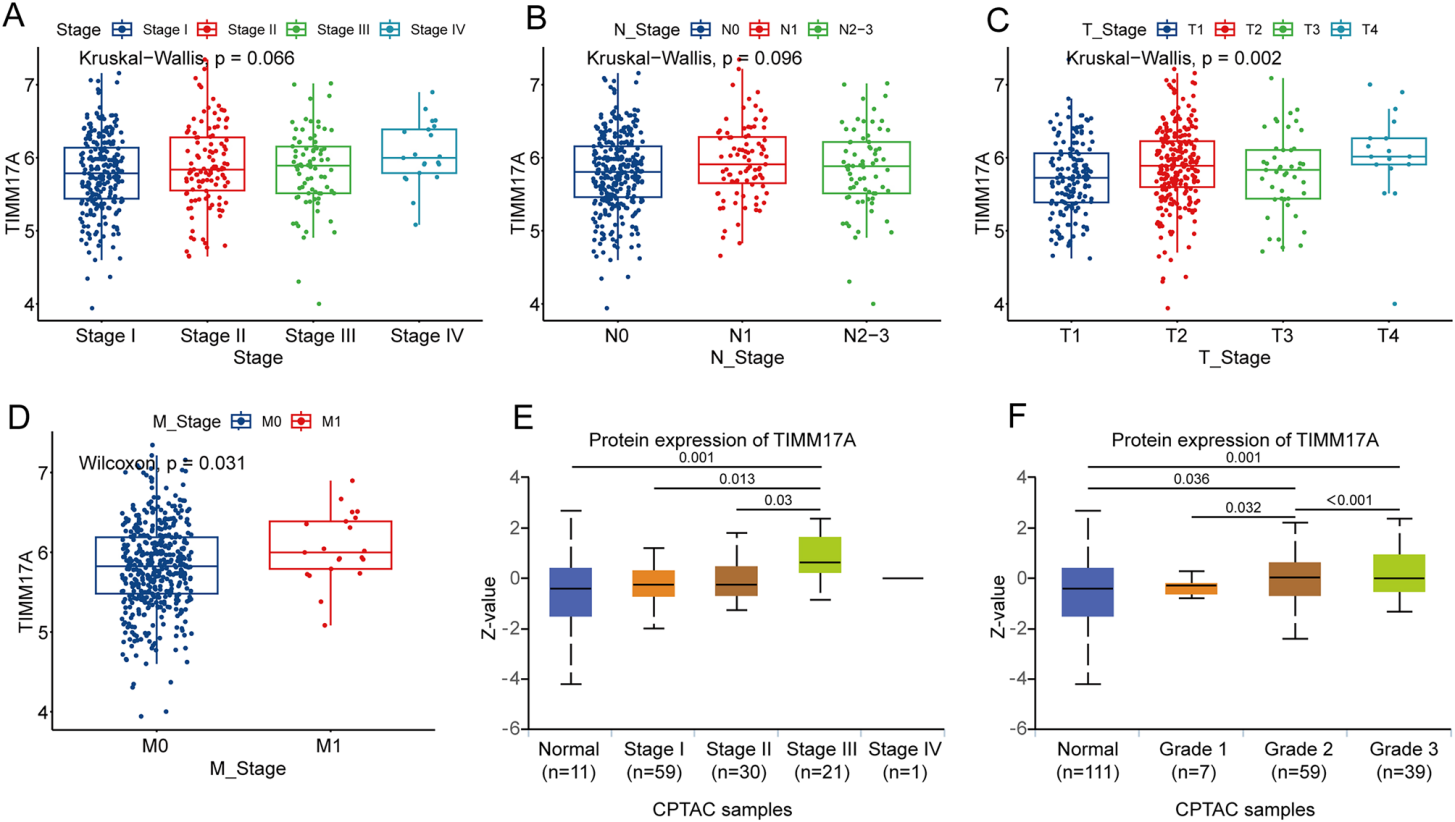
Relationship between TIMM17A expression and clinical features in patients with LUAD. (**A**) TIMM17A expression is not significantly different between different stages of LUAD patients. (**B**) No significant difference in TIMM17A expression was observed between N stages. (**C**) TIMM17A expression significantly increases with T stage. (**D**) In patients with M1 stage LUAD, TIMM17A expression is significantly higher than in those with M0 stage. (**E**) TIMM17A expression is substantially higher in Stage III LUAD samples compared to those in normal, Stage I, and Stage II groups, as confirmed by the CPTAC database. (**F**) The expression of TIMM17A protein increases significantly with Grade stage.

Our investigation explored the TCGA LUAD dataset to examine the complex relationship between TIMM17A expression and the clinical features of LUAD patients. While there was no marked difference in TIMM17A expression across different tumor stages (Fig. 4A) and N-stage (Fig. 4B), a notable increase in TIMM17A expression was observed across LUAD Stage I-IV. Significantly, TIMM17A levels were higher in patients with lymph node metastasis compared to those without. In terms of T-stage, TIMM17A expression notably escalated with advancing disease stage, a trend that was statistically significant (Fig. 4C). Contrarily, TIMM17A expression was strikingly higher in patients with M1 stage compared to M0 stage (Fig. 4D). Additionally, in Stage III LUAD samples, TIMM17A expression significantly exceeded that in normal, Stage I, and Stage II groups, as confirmed by the CPTAC database (Fig. 4E). Furthermore, in our analysis of tumor grade, TIMM17A protein expression substantially increased with advancing grade (Fig. 4F). In summation, the intricacies of the intricately woven relationship between TIMM17A and the malignancy and prognosis of LUAD, render TIMM17A a potential harbinger of LUAD prognosis.

### Univariate and multivariate analysis of TIMM17A in LUAD

**Figure 5:**
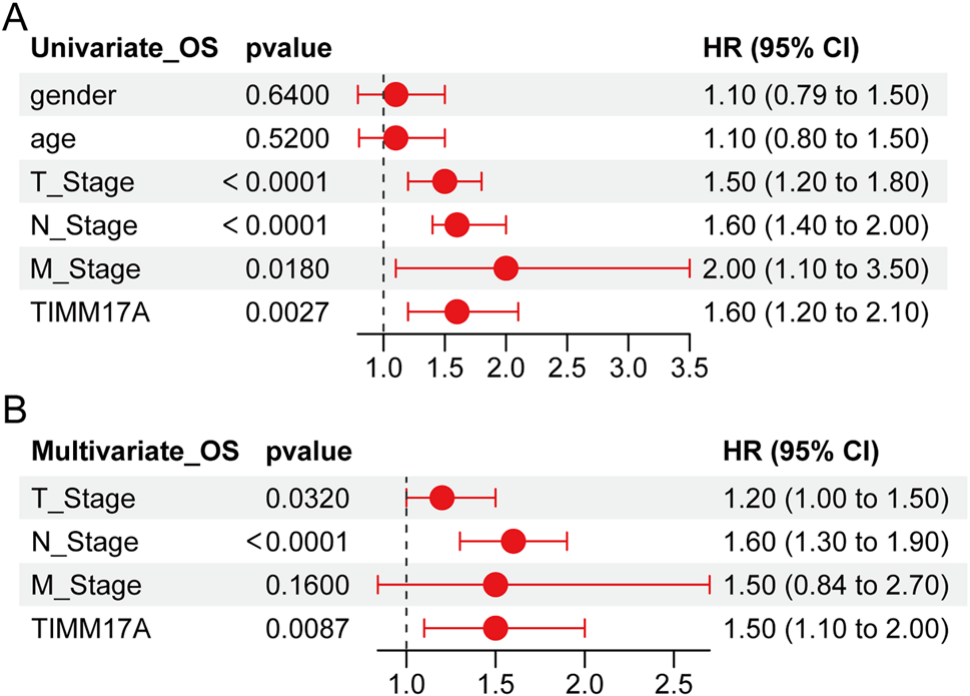
Univariate and multivariate analysis of TIMM17A in LUAD. (**A**) The results of the one-way Cox method show that T-stage, N-stage, M-stage, and TIMM17A are all significant prognostic factors. (**B**) The multifactorial Cox analysis further highlights the independent prognostic significance of TIMM17A.

In order to thoroughly investigate the multifaceted and complex nature of LUAD prognosis, our team engaged in a rigorous and comprehensive analysis utilizing the one-way Cox method. We meticulously examined a range of potentially impactful factors, including gender, age, T-stage, N-stage, M-stage, and TIMM17A. The results of this exhaustive analysis revealed a number of key findings, most notably the fact that T-stage, N-stage, M-stage, and TIMM17A all played a significant role in the prognosis of LUAD (Fig. 5A). However, we did not stop there. In order to more fully verify the independence of these factors, and to gain a more nuanced understanding of their individual contributions to the overall picture, we conducted a multifactorial Cox analysis. Our efforts bore fruit, as this analysis revealed TIMM17A to be one of the truly independent prognostic factors for LUAD, as evinced by the striking and revealing Fig. 5B. The implications of this groundbreaking finding cannot be overstated. Indeed, it is abundantly clear that the role of TIMM17A in the prognosis of LUAD is of paramount importance, and that it has significant implications for the development of effective treatments and prognostic strategies moving forward. In light of these exciting and novel findings, we believe that TIMM17A should be considered as a leading candidate for further exploration as a potential marker for the treatment and prognosis of LUAD.

### GSEA of TIMM17A in LUAD

**Figure 6:**
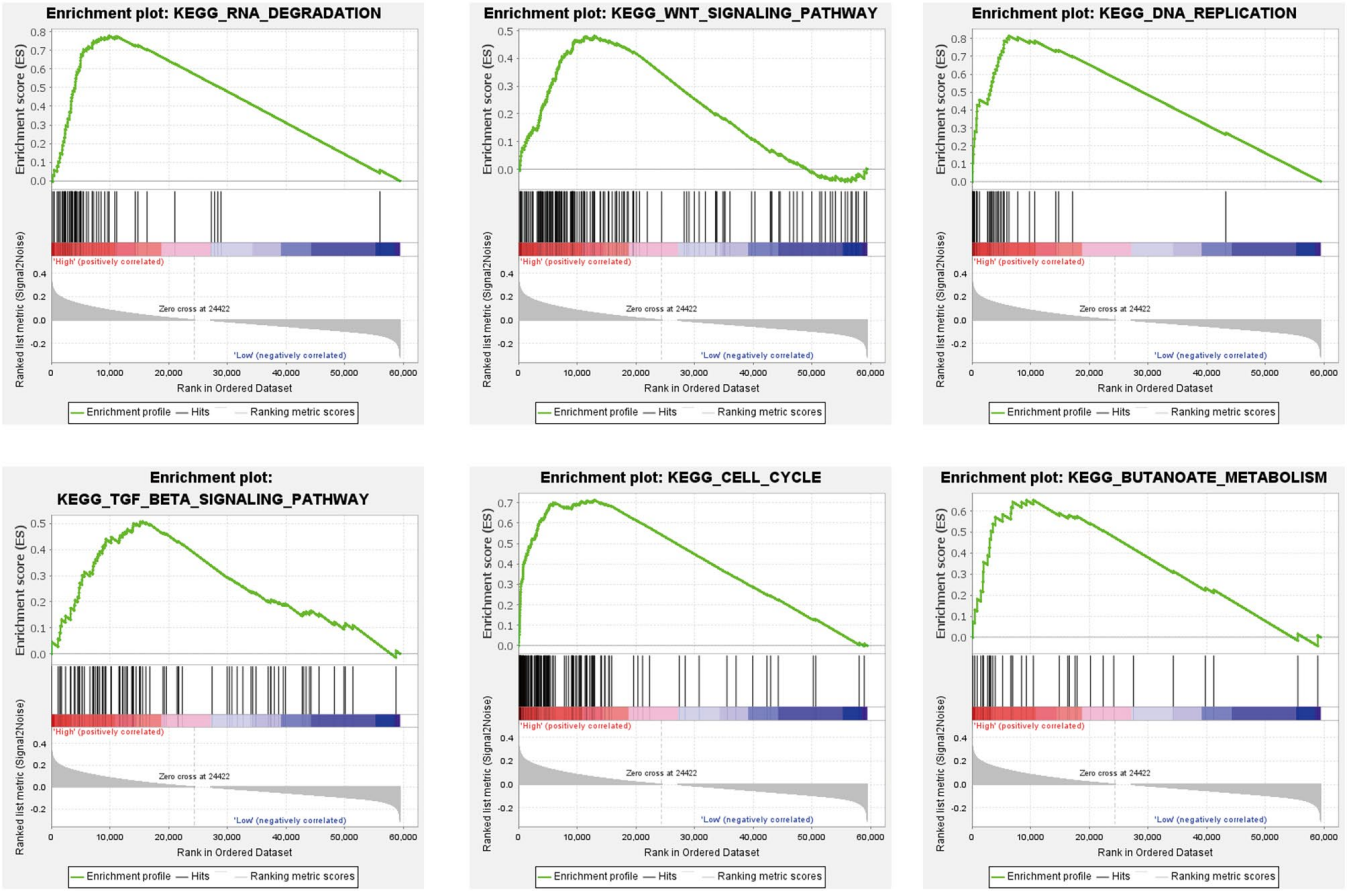
GSEA analysis of TIMM17A in LUAD. The GSEA analysis shows that TIMM17A is associated with several signaling pathways, including Butanoate metabolism, Cell cycle, DNA replication, RNA degradation, TGF beta signaling pathway, and WNT signaling pathway.

In our endeavor to decipher the complex role of TIMM17A in regulating LUAD staging, we employed GSEA. This meticulous analysis revealed a significant association between TIMM17A expression and various biological processes, including Butanoate_metabolism, Cell_cycle, DNA_replication, RNA_degradation, TGF_beta signaling_pathway, and WNT_signaling_pathway, among others (Fig. 6A, Table 1). This finding is a pivotal contribution to our understanding of how TIMM17A influences the diverse biological pathways integral to LUAD staging. The complexity of these interactions is immense, yet our use of GSEA has enabled us to make substantial strides in elucidating this intricate network.

**Table 1:** Gene set enrichment analysis results.

MSigDB collection	Gene set name	NES	NOM	FDR
			*p*-val	q-val
c2.cp.kegg.v7.5.1.symbols.gmt	KEGG_RNA_DEGRADATION	2.65	< 0.001	< 0.001
	KEGG_DNA_REPLICATION	1.91	< 0.001	0.012
	KEGG_CELL_CYCLE	2.09	0.002	0.002
	KEGG_BUTANOATE_METABOLISM	1.92	0.004	0.011
	KEGG_TGF_BETA_SIGNALING_PATHWAY	1.70	0.028	0.046
	KEGG_WNT_SIGNALING_PATHWAY	1.69	0.030	0.049

### Nomogram model

**Figure 7:**
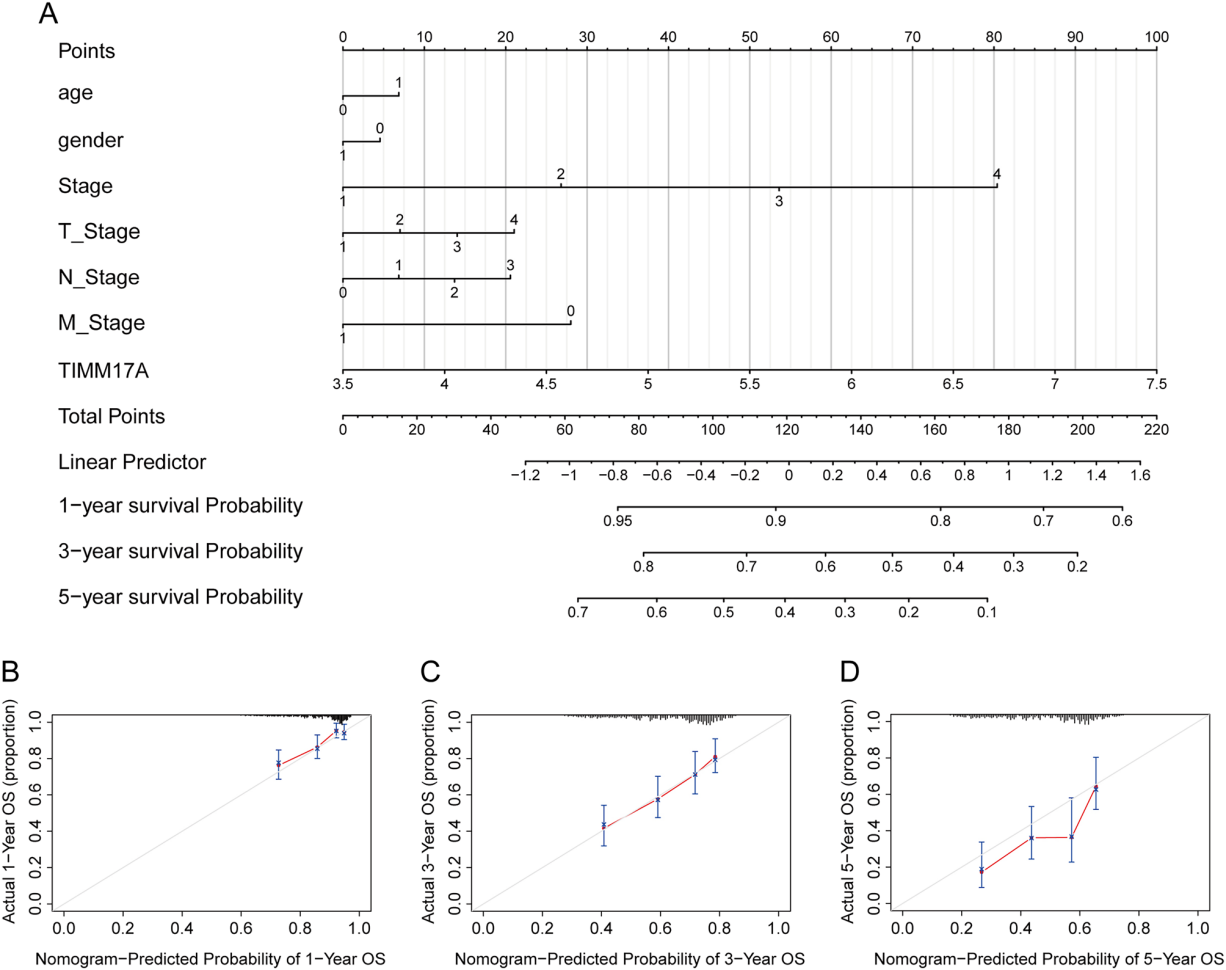
Nomogram model. (**A**) After incorporating TIMM17A into the Nomogram model, the effectiveness of the model in prognosticating LUAD patients was examined. (**B**–**D**) Calibration curves for the Nomogram model at 1.3.5 years.

After utilizing TIMM17A in the Nomogram model construction, we proceeded to examine its effectiveness in prognosticating LUAD patients, as illustrated in Fig. 7A. Notably, the results of our calibration efforts revealed a remarkable degree of predictive accuracy (Fig. 7B–D). It is thus safe to assert that the model is a highly reliable tool for forecasting the prognosis of patients suffering from LUAD.

### The prognostic and in vitro cell experiment of TIMM17A in LUAD

**Figure 8:**
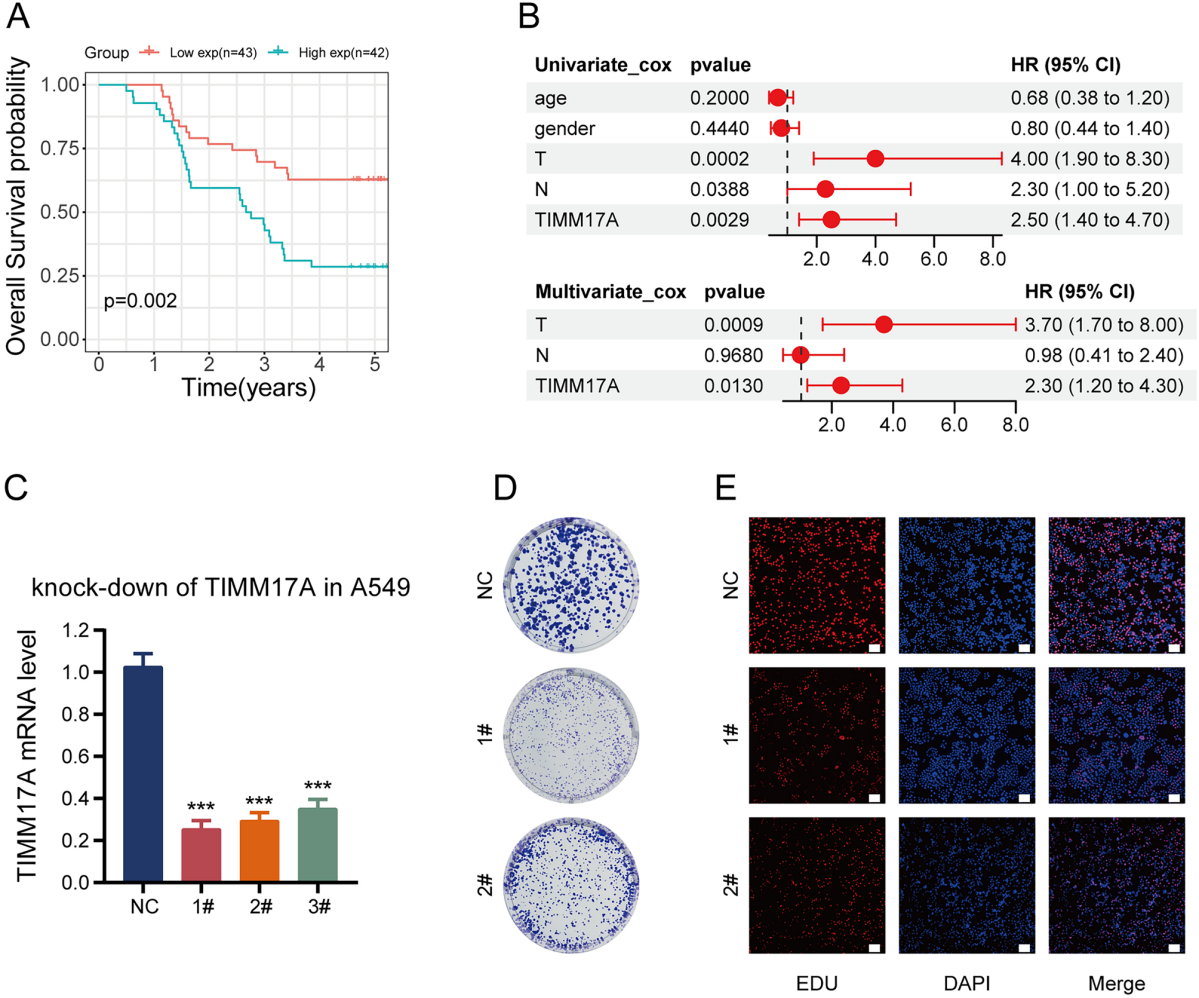
Prognostic and In vitro Cell Experiment of TIMM17A in LUAD. (**A**) Kaplan–Meier survival curve analysis of TIMM17A in a cohort of 85 LUAD cases. (**B**) Univariate Cox and multivariate Cox analyses of TIMM17A in the 85 LUAD cases. (**C**) qRT-PCR analysis showing the knockout efficiency of TIMM17A in A549 cells. (**D**) Clone formation experiment following TIMM17A knockdown in A549 cells. (**E**) Edu cell proliferation experiment after TIMM17A knockdown in A549 cells; scale bar: 100 μm.

In our final analysis, we conducted a logical examination of the pathological characteristics and expression of TIMM17A in 85 cases of LUAD patients, revealing a correlation between TIMM17A and LUAD T stage, N stage, and overall stage (Table 2). Kaplan–Meier survival curves indicated a significant association between high TIMM17A expression and adverse prognosis (Fig. 8A). Univariate Cox regression demonstrated that T stage, N stage, and TIMM17A may serve as adverse prognostic factors for LUAD. Further multivariate Cox analysis identified TIMM17A as an independent prognostic factor for LUAD, particularly associated with T stage (Fig. 8B). To delve deeper into the functional significance of TIMM17A, we performed TIMM17A gene knockdown experiments in A549 cells (Fig. 8C). Clone formation assays and Edu cell proliferation experiments following TIMM17A knockdown demonstrated a significant suppression of A549 cell proliferation (Figs. 8D,E). In summary, the inhibition of A549 cell proliferation upon TIMM17A knockdown suggests its crucial role in the development of LUAD.

**Table 2:** Correlation between TIMM17A expression in LUAD tissues and clinicopathological features of LUAD patients.

Clinicopathological	n = 85	TIMM17A expression		*p* value
parameter		Low 43	High 42	
Gender				0.425
Male	34	19	15	
Female	51	24	27	
Age (years)				0.235
≤ 60	39	17	22	
> 60	46	26	20	
TNM stage				0.012
I + II	38	25	13	
III	47	18	29	
T-stage				0.018
T1 + T2	33	22	11	
T3 + T4	52	21	31	
N-stage				0.001
N0	21	17	4	
N1 + N2 + N3	64	26	38

## Discussion

LUAD, a malignancy originating from glandular epithelium, stands as one of the most prevalent cancers globally^27^. In spite of notable progress in contemporary treatment modalities, LUAD remains a major cause of mortality, underscoring the imperative need for continued research into innovative therapeutic targets and prognostic biomarkers^28^. The pursuit of molecular markers that can accurately reflect the complex nature of LUAD, coupled with the elucidation of their intricate associations with the clinical characteristics of affected patients, has emerged as a critical area of focus in the ongoing battle against this formidable disease^29,30^.

TIMM17A has been increasingly recognized as a key player in tumorigenesis, with its aberrant expression linked to the progression and prognosis of various cancers. The role of TIMM17A in breast cancer has been extensively studied. Overexpression of TIMM17A in breast cancer consistently correlates with accelerated disease progression, decreased OS, and reduced distant metastasis survival, suggesting its potential as a therapeutic target^12^. In invasive and triple-negative breast cancer (TNBC), TIMM17A has been identified as a key target gene of miR-24-2 and miR-133b, with its low expression associated with improved survival, indicating diagnostic and therapeutic potential^31,32^. High expression of TIMM17A mRNA in breast cancer is linked to adverse pathological parameters and poor clinical outcomes, positioning TIMM17A as an independent predictor of OS and a potential prognostic marker^13^. In fibrolamellar hepatocellular carcinoma (FLC), including primary and metastatic tumors, TIMM17A is frequently overexpressed on the 1q chromosome, suggesting its potential role as an oncogene in this rare subtype of liver cancer^33^. However, research on TIMM17A in LUAD is relatively limited.

Therefore, we embarked on an in-depth investigation into the expression of the TIMM17A gene in LUAD and its potential significance in the prognosis and clinical features of the disease. Through the analysis of TIMM17A mRNA expression in the TCGA LUAD dataset and combining protein expression data from CPTAC and HPA databases, we concluded that TIMM17A is aberrantly upregulated in LUAD. To validate this finding, we conducted qRT-PCR verification in 85 LUAD patients, and the results were consistent with the database expression, strengthening our observations. Regarding the relationship between TIMM17A and the prognosis of LUAD patients, we employed Kaplan–Meier survival curves and Cox regression analysis, revealing a correlation between high TIMM17A expression and adverse outcomes. Furthermore, in the TCGA LUAD dataset, TIMM17A showed associations with pathological features of LUAD, and logical analysis of TIMM17A expression and pathological features in 85 LUAD patients indicated correlations with T staging, N staging, and Stage grading. These results further underscored the potential role of TIMM17A in LUAD. To comprehensively understand the impact of TIMM17A on LUAD, we conducted GSEA analysis, revealing TIMM17A enrichment in various critical biological processes and pathways, such as Butanoate_metabolism, Cell_cycle, DNA_replication, RNA_degradation, TGF_beta_signaling_pathway, and WNT_signaling_pathway. These findings suggest that TIMM17A may participate in influencing the progression of LUAD through these pathways. In addition, considering the multifactorial Cox analysis showing the correlation between TIMM17A and T staging in 85 LUAD patients, we performed TIMM17A mRNA knockdown experiments to validate this association. The results demonstrated that the knockdown of TIMM17A inhibited the proliferation ability of A549 cells, further confirming the crucial role of TIMM17A in the development of LUAD.

While our study presents significant insights, it is important to acknowledge certain limitations. First, the reliance on bioinformatics analysis and cellular experiments in this research underlines the need for additional validation through clinical samples^30^. Second, the specific molecular mechanisms underpinning TIMM17A's role remain unexplored, warranting further experimental investigation for a more comprehensive understanding. Lastly, although this study highlights TIMM17A's potential relevance in LUAD, the practicality of its application in clinical settings necessitates more extensive prospective research for definitive validation^34^.

## Conclusions

Overall, our bioinformatics analysis substantiates the role of TIMM17A as a promising diagnostic biomarker and therapeutic target in LUAD. The experimental findings underscore TIMM17A's critical involvement in LUAD pathogenesis, especially its influence on cellular proliferation. Furthermore, this study offers an initial insight into the role of TIMM17A in LUAD, laying a solid foundation for future research endeavors to expand upon.

## Data Availability

The primary and processed data used in our analysis can be downloaded from the TCGA LUAD publication page (https://portal.gdc.cancer.gov/).
